# Investigation of the Antifungal Activity and Mode of Action of *Thymus vulgaris*, *Citrus limonum*, *Pelargonium graveolens*, *Cinnamomum cassia*, *Ocimum basilicum*, and *Eugenia caryophyllus* Essential Oils

**DOI:** 10.3390/molecules23051116

**Published:** 2018-05-08

**Authors:** Katarzyna Gucwa, Sławomir Milewski, Tomasz Dymerski, Piotr Szweda

**Affiliations:** 1 Department of Pharmaceutical Technology and Biochemistry, Faculty of Chemistry, Gdańsk University of Technology, Narutowicza 11/12 Str., 80-233 Gdańsk, Poland; katarzyna.gucwa@pg.edu.pl (K.G.); slawomir.milewski@pg.edu.pl (S.M.); 2 Department of Analytical Chemistry, Faculty of Chemistry, Gdańsk University of Technology, Narutowicza 11/12 Str., 80-233 Gdańsk, Poland; tomasz.dymerski@pg.edu.pl

**Keywords:** anti-*Candida* activity, essential oils, modes of action, synergism, GC × GC MS analysis

## Abstract

The antimicrobial activity of plant oils and extracts has been recognized for many years. In this study the activity of *Thymus vulgaris*, *Citrus limonum*, *Pelargonium graveolens*, *Cinnamomum cassia*, *Ocimum* *basilicum*, and *Eugenia caryophyllus* essential oils (EOs) distributed by Pollena Aroma (Nowy Dwór Mazowiecki, Poland) was investigated against a group of 183 clinical isolates of *C. albicans* and 76 isolates of *C. glabrata*. All of the oils exhibited both fungistatic and fungicidal activity toward *C. albicans* and *C. glabrata* isolates. The highest activity was observed for cinnamon oil, with MIC (Minimum Inhibitory Concentration) values in the range 0.002–0.125% (*v*/*v*). The MIC values of the rest of the oils were in the range 0.005% (or less) to 2.5% (*v*/*v*). In most cases MFC (Minimum Fungicidal Concentration) values were equal to MIC or twice as high. Additionally, we examined the mode of action of selected EOs. The effect on cell wall components could not be clearly proved. Three of the tested EOs (thyme, lemon, and clove) affected cell membranes. At the same time, thyme, cinnamon, and clove oil influenced potassium ion efflux, which was not seen in the case of lemon oil. All of the tested oils demonstrated the ability to inhibit the transition of yeast to mycelium form, but the effect was the lowest in the case of cinnamon oil.

## 1. Introduction

*Candida albicans* is a common opportunistic fungal pathogen that inhabits the bodies of healthy individuals. Although other species of non-*albicans Candida*, such as *C. tropicalis*, *C. parapsilosis*, *C. krusei*, and *C. lusitaniae*, have shown an increased incidence of nosocomial infections, *C. glabrata* is still considered the most common non-*albicans* isolated *Candida* species [1,2,3]. In immunocompromised patients, both *C. albicans* and *C. glabrata* can cause superficial mucosal infection, such as oral thrush and vaginitis, as well as potentially life-threatening systemic disorders [4]. High-risk groups include organ transplant recipients, cancer patients receiving chemotherapy, and people with HIV/AIDS. In the last few years, *Candida* infections have occurred more frequently, with high mortality rates, and have been recognized as one of the most significant causes of hospital-acquired infections [5].

Despite the high incidence and the severity of *Candida* infections, treatments are still limited and insufficient. In the treatment of fungal infections there are only a few drug classes available: polyenes, triazole derivatives, echinocandins, allylamines, and flucytosine [6]. Nevertheless, none of them meets all the expectations (especially regarding low toxicity to patients, convenience of administration, and a low possibility of resistance acquiring).

Therefore, new therapeutic alternatives based on exploitation of natural resources have been intensively investigated recently [7]. Essential oils (EOs) have gained increased interest due to their antiseptic and antimicrobial activity. Many researchers have reported on their antibacterial [8], antifungal [9], anti-parasitic [10], and antiviral activity [11]. EOs are rich mixtures of chemical compounds belonging to different chemical families, including terpenes, aldehydes, alcohols, esters, phenols, ethers, and ketones. Most essential oils are composed of terpenes, terpenoids, and other aromatic and aliphatic constituents with low molecular weights. Terpenes are a class of natural substances of vegetable origin formed by the condensation of isoprene units (C_5_H_8_) and are classified as monoterpenes (C_10_), the most representative molecules, and sesquiterpenes (C_15_) [12]. Their derivatives containing oxygen are called terpenoids. Usually, the chemical characterization of many essential oils reveals the presence of only 2–3 major components at a fairly high concentration (20–70%) compared to other components present in trace amounts [13].

The mechanism of antimicrobial action of EOs is complex and depends on their chemical composition and the quantity of the major single compounds. The antifungal mechanism of action of EOs is similar for antibacterial. Many reports revealed that constituents of EOs mixture cause cell membrane damage; moreover, they influence many other cellular activities including energy production [8]. The antimicrobial effect may be linked to reduced membrane potentials, the disruption of proton pumps, and the depletion of the ATP [14]. The effect of EOs activity is also the coagulation of cell content, cytoplasm leakage, and finally cell apoptosis or necrosis, leading to cell death [8].

The present study aimed to select the most potent EOs and to evaluate their activity on a large group of *C. albicans* and *C. glabrata* clinical isolates. Furthermore, a synergistic effect of the EOs with commonly used antimycotics was investigated, as well as the mechanism of action of the selected EOs.

## 2. Results

### 2.1. Minimum Inhibitory Concentrations (MIC) and Minimum Fungicidal Concentrations (MFC)

The antifungal activity was determined in a buffered to pH 7.0 RPMI-1640 medium supplemented with 2% glucose, which is recommended by CLSI for determining the activity of antifungal agents. The composition of this medium corresponds to the physiological conditions in the host’s organism (human or animals). Our preliminary research of the antifungal potential of a set of 37 essential oils revealed that oils isolated from *Thymus vulgaris*, *Citrus limonum*, *Pelargonium graveolens*, *Cinnamomum kasia*, *Ocimum basilicum*, and *Eugenia caryophyllus* exhibited the highest activity [15]. Herein we evaluated their effectiveness against the group of 183 clinical isolates of *C. albicans* and 76 clinical isolates of *C. glabrata* (Table 1, Table 2, Table 3 and Table 4). Moreover, we determined the chemical composition of all of these products.

Among all tested EOs, cinnamon oil exhibited the highest antifungal activity against isolates of both species with MIC and MFC values in the range of 0.002 (or less) to 0.125% (*v*/*v*), followed by oil obtained from *Thymus vulgaris* (MIC and MFC values ≤ 2.5% (*v*/*v*) against all strains tested). Within the tested range of concentration—up to 2.5% (*v*/*v*) the single strains resistant to the activity of other oils were identified (Table 1 and Table 2). In the case of cinnamon oil most of the tested isolates of *C. albicans* (*n* = 114; 62.3%) revealed MIC below the concentration 0.002% (*v*/*v*) and respectively for *C. glabrata* that was 56.6% (*n* = 43). The modal of MIC values for thyme oil was 0.08% (*v*/*v*), which was observed for 31 isolates (16.9%), however the growth of 30 isolates was inhibited at the concentration of this product lower than 0.005% (*v*/*v*). The most frequent MIC value of geranium and clove oil was 0.16% and in the case of lemon and basil oils 0.313% (*C. albicans*). In the case of *C. glabrata* that was 0.005% for lemon oil, 0.16% for thyme, geranium, and clove and 0.313% for basil oil. Considering fungicidal activity, the MFC concentration was mostly equal to or twice the MIC value (Table 3 and Table 4). Only in some cases MFC was much higher than MIC.

### 2.2. Time Kill Assay

**Figure 1:**
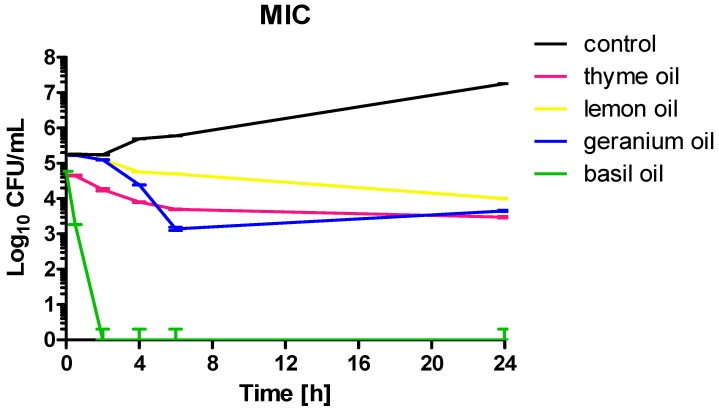
Kinetic of *C. albicans* ATCC 10231 growth under the influence of EOs in the following concentrations: thyme—0.04%, lemon 0.16%, geranium—0.16%, basil 0.16%.

**Figure 2:**
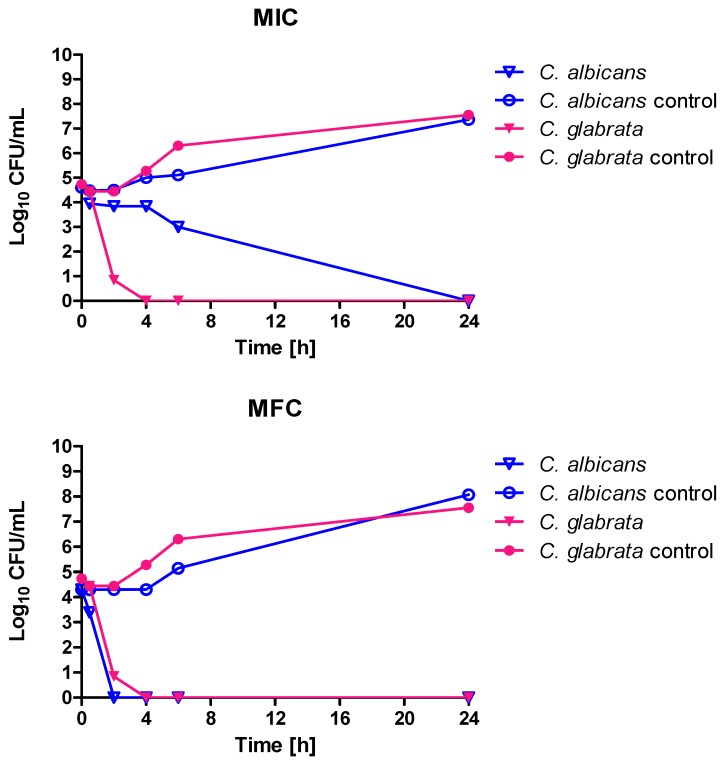
Kinetic of *C. albicans* ATCC 10231 and *C. glabrata* DSM 11226 growth under the influence of cinnamon oil in the concentrations equal to 0.016% (*v*/*v*) (MIC) or 0.031% (*v*/*v*) (MFC).

*C. albicans* ATCC 10231 cells were treated with EOs at a concentration equal to MIC as follows: thyme—0.04%, lemon 0.16%, geranium—0.16%, basil 0.16%, clove—0.625%. Figure 1 shows that incubation with four of these products resulted in fungistatic effect, with a significant reduction in cell numbers after 6 and 24 h of at least two logarithmic rows. Much better results were obtained for basil oil, where 2 h incubation reduced the number of living cells to nearly zero. That means that for this strain the MIC and MFC values of basil oil were equal. For cinnamon oil separate analysis, with concentrations of 1 × MIC and 1 × MFC for two *Candida* species, was performed. The obtained results clearly indicate that both applied concentrations caused a fungicidal effect. However, for *C. albicans* at a concentration equal to 1 × MIC a 24-h incubation was required. In other cases, a four-hour incubation was sufficient for total viability decrease (Figure 2).

### 2.3. Sorbitol Assay Effect of Essential Oils on the Cell Wall of C. albicans and C. glabrata

MIC values for *C. albicans* and *C. glabrata* were checked in the presence or absence of 0.8 M sorbitol as an osmotic protectant in the medium. In the case of lemon and geranium oils, the MIC values stayed unchanged what suggests lack of their influence on cell wall structure (Table 5). For basil oil, the MIC value in the presence of osmoprotectant was doubled for both tested species, indicating that cell wall may be a possible molecular target for this EO. In the presence of sorbitol, MIC values for thyme, clove, and, in particular, cinnamon oil were significantly decreased.

Additionally, an assay comparing cinnamon oil and cinnamaldehyde MIC values in the absence or presence of osmoprotectant (sorbitol or glycerol) was carried out (Table 6). The assay was performed on two reference strains and two clinical isolates (*C. albicans* 412 and *C. glabrata* 342, both isolated from Children’s Memorial Health Institute’s patients). MIC values for cinnamon oil and cinnamaldehyde were in the range 0.008–0.031% (*v*/*v*), while in the medium enriched with sorbitol these values decreased at least 4-fold, similar to the medium containing the added glycerol.

### 2.4. Ergosterol Binding Assay

Exogenous source of ergosterol in the medium may increase the MIC value for compounds that target this sterol in the cell membrane. In our study MIC values were significantly increased in the case of thyme and lemon oil in both species and slightly for clove oil. In the case of cinnamon and basil oil, the MIC values were unchanged or nearly unchanged in the ergosterol-containing medium. Amphotericin B, an agent known to act on ergosterol in the membrane, was used as a positive control (Table 5).

### 2.5. Potassium Ion Efflux

**Figure 3:**
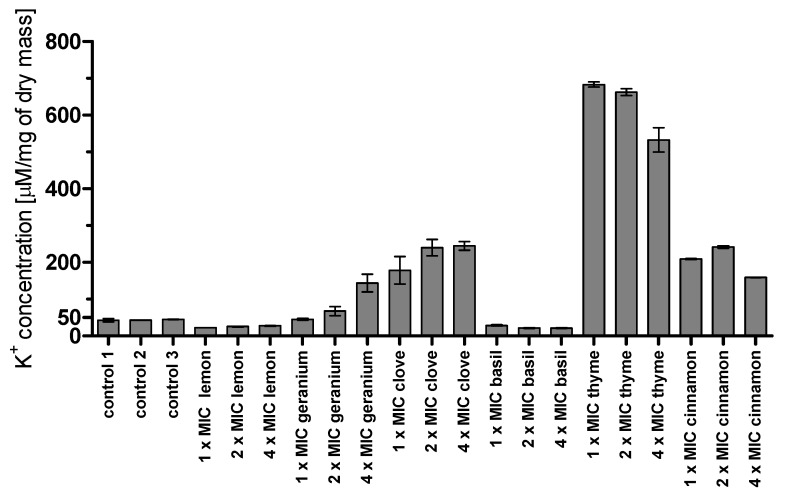
Potassium ion efflux induced by EOs at three concentrations 1 × MIC, 2 × MIC, and 4 × MIC. Error bars indicate uncertainty of measurement.

The efflux of potassium ions to a potassium-free medium is a common response of the cells to the presence of some compounds like essential oils. As Figure 3 shows, at the concentrations of agents used, nearly no potassium efflux was detected in the presence of basil and lemon oil (the heights of the bars are comparable to the heights of the cells suspension bars). The highest efflux of potassium was observed for thyme oil, followed by clove and cinnamon oil. A little efflux of potassium was detected in the case of geranium oil, but only at the highest concentration. Additionally, in the case of thyme and cinnamon oil, there was no correlation between the potassium efflux and the concentration of EO used.

### 2.6. Yeast to Mycelia Morphological Transition

A control sample of *C. albicans* ATCC 10231 not exposed to the action of essential oil grown for 2 h in hypha-inducing Lee medium resulted in 95% of mycelium forms. Cultures of the same strain incubated with selected EOs at concentrations equal to 1 × MIC revealed nearly now hypha forms, except of the sample that was under the influence of basil oil, where some mycelium forms were still visible (Table 7). Cultures were also treated with EOs for 24 h at concentrations equal to ½ × MIC (in order to use the highest available concentration and not inhibit growth at the same time). The percentage of mycelium forms in the control significantly declined, comparable to the sample treated with cinnamon oil. Hypha forms were also still observed in the sample with added basil oil.

### 2.7. Synergism between EOs and Antifungal Drugs

**Figure 4:**
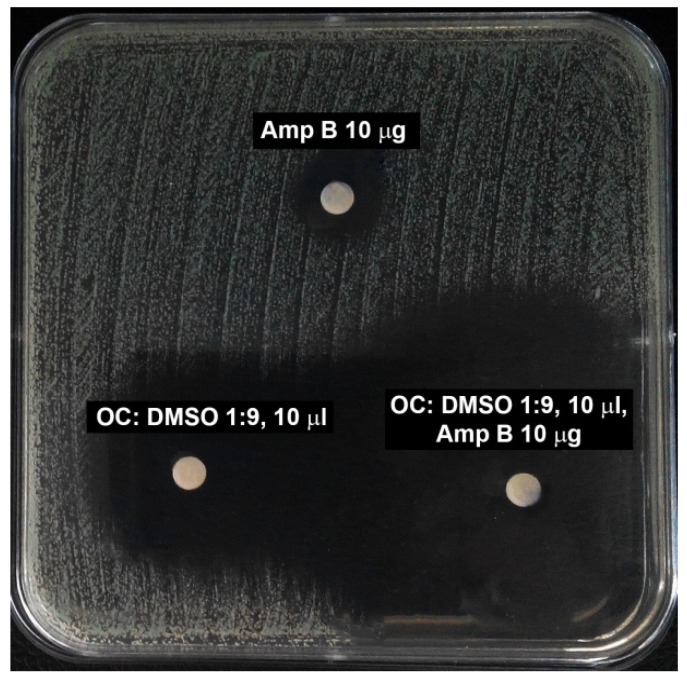
Synergistic action of amphotericin B with cinnamon oil (upper disc 10 μg of amphotericin B alone, bottom left 10 μL of cinnamon oil alone diluted 10 times with DMSO (dimethyl sulfoxide), bottom right AmB and EO in combination). DMSO alone does not show the inhibitory zone.

Studies revealed nearly no synergistic interactions between antifungal drugs and the essential oils tested. The only example of possible synergism was between amphotericin B and geranium oil. The MIC of amphotericin B alone was evaluated at 0.125 μg/mL, while the MIC of EO was 0.08%. Inhibitory concentrations of the compounds in combination were as follows: 0.031 μg/mL for AmB and 0.02% for geranium oil. According to the checkerboard method, ∑FIC = 0.5, so the interaction can be considered as synergistic. Additionally, using the disc-diffusion method a synergistic effect of AmB and cinnamon oil was observed (Figure 4).

### 2.8. Chemical Compositions of the Most Active Essential Oils

The composition analysis of six EOs has been performed by GC × GC mass spectrometry. A quantitative analysis has been carried out by peak area normalization measurements without correction factors as percentages of each component (Table 8). The highest diversity in chemical composition was marked for geranium oil, but the content of any particular component did not exceed 20%. The smallest number of components was seen for clove oil, with a predominance of eugenol (68.24%). Some monoterpenes were common for most of the tested oils (camphene, cymene, myrcene, pinene, and terpinene). The most active cinnamon oil contained high concentration of cinnamaldehyde (42.79%), while lemon oil contained the highest amount of citral (53.85%).

## 3. Discussion

Historically, many plant oils and extracts have been used as topical antiseptics, or have been reported to have antimicrobial properties [16]. Thus, it is very important to investigate scientifically those plants that have been used in traditional medicines as potential sources of novel antimicrobial compounds. Beside the high antimicrobial potential, one important advantage of essential oils is the complex mechanism of action arising from a rich mixture composition.

In this paper we showed the high antifungal activity of *Thymus vulgaris*, *Citrus limonum*, *Pelargonium graveolens*, *Cinnamomum cassia*, *Ocimum basilicum*, and *Eugenia caryophyllus* essential oils. The maximum concentration that prevented the growth of most *Candida* isolates was established as 1.25% (with some exceptions for lemon and clove oil). This allows for their potential use in pharmaceutical preparations for external application or inhalation, as using a 2% essential oil dilution is generally considered a safe guideline for the topical application of essential oils on adults [17]. Cinnamon, thyme, geranium, and basil oils inhibited the growth of all strains tested, up to the concentration of 1.25%. In the case of lemon oil, only seven out of 183 *C. albicans* isolates had an MIC value higher than the cutoff value of 1.25%. Quite a similar situation was observed for clove oil: MIC = 2.5% (*v*/*v*) was found for eight *C. albicans* isolates and one *C. glabrata*. Comparing the differences in activity of oils against two *Candida* species, no significant differences were observed except for lemon oil, which appeared to be more active toward *C. glabrata* clinical isolates (for example, 35.5% of isolates revealed MIC values below 0.005%; for *C. albicans* that was 11.5%). A great activity was established in the case of cinnamon oil. The concentration inhibiting the growth of all strains was not higher than 0.125% (*v*/*v*). The vast majority of isolates revealed MIC values for this oil below 0.021 mg/mL (0.002%). Other investigators recorded similar results. For instance, Wang and coworkers reported that the average MIC value of cinnamon oil tested toward *C. albicans* clinical isolates was 0.064 mg/mL (MIC range 0.064–0.515 mg/mL) [18]. The research group of Pires showed the anticandidal activity of cinnamon oil against *C. orthopsilosis* and *C. parapsilosis* (MIC equal to 0.25 and 0.50 mg/mL, respectively) [19]. High antifungal activity against six *Candida* isolates was also reported by Brochot (MIC in the range 0.01–0.05% (*v*/*v*)) [20]. The activity of cinnamon oil toward bacteria seems to be limited compared to fungi, e.g., the MIC values for *S. aureus* and *E. coli* reported by Zhang were both 1 mg/mL, while MBC values were equal to 4 mg/mL [21]. The explanation of this fact is connected to the molecular targets for cinnamaldehyde, which are glukan and chitin synthases absent in bacteria. On the other hand, other researchers have shown that cinnamon oil may cause other cellular effects like leakage of small electrolytes, proteins, and nucleic acids [21]. Nowatarska reports that cinnamon oil decreases the intracellular adenosine triphosphate concentration [22]. Additionally, the results of Clemente indicate that cinnamon oil produces protrusions and aggregation of cells [23]. In our opinion, all the further destructive effects of cinnamon oil toward pathogen cells are a result of its complex composition, in which remaining compounds have supplementary or synergistic activity. However, cinnamon aldehyde is crucial to the antifungal activity of this product. The GC analysis revealed that the investigated oil contained 42.79% of this component.

The highest thyme oil MIC concentration established for both *C. glabrata* and *C. albicans* was 5.731 mg/mL (0.625% (*v*/*v*)), but predominant values were 0.734 mg/mL (0.08% (*v*/*v*)) or less than 0.046 mg/mL (0.005% (*v*/*v*)). These results are in agreement with those obtained by other investigators. High anticandidal activity of thyme oil (*Thymus capitatus*) was reported by Sakkas and coworkers (MIC 0.125 or 0.25% (*v*/*v*)) [24]. The effectiveness of *Thymus vulgaris* EO toward *C. albicans* was also confirmed by Fani [25]. Antimicrobial activity (mainly toward bacteria) of *Thymus vulgaris* (thymol chemotype), *Thymus zygis* subsp. *gracilis* (thymol and two linalool chemotypes), and *Thymus hyemalis* Lange (thymol, thymol/linalool and carvacrol chemotypes) essential oils extracted from seven plants cultivated in Murcia (Spain) was reported by Rota and coworkers [26]. Jamali tested the anticandidal activity of seven *Thymus* species: *Thymus broussonetii*, *T. ciliates*, *T. leptobotrys*, *T. maroccanus*, *T. pallidus*, *T. satureioides*, and *T. serpyllum* collected from different natural regions in southern and southwestern Morocco. He reported high antifungal activity toward four *Candida* species (*C. albicans*, *C. krusei*, *C. glabrata*, and *C. parapsilosis*) for EOs rich in thymol or carvacrol (MIC 0.43–0.9 mg/mL). *T. serpyllum* EO, in which the predominant compound detected was linalyl acetate (52.2%), had the lowest anticandidal activity (MIC in the range 3.52–7.05 mg/mL) [27]. The investigated in our studies thyme oil contained carvacrol and thymol at concentrations comparable to the product investigated by Jamali—0.42 and 1.75% of the sum of peaks area respectively. However, the dominant components of the product were myrcene (34.35% of area under the peak) and terpinene (46.55% of area under the peak). High activity of the tested EOs was also confirmed in a time kill assay. Most EOs in MIC concentration resulted in fungistatic effect (after 6 h incubation, the number of viable cell was stable and at least three logarithmic rows lower than in the control). When MIC and MFC values are equal, we observed very rapid viable cell count reduction (like in the case of basil oil). The usage of cinnamon oil in MFC concentration resulted in a reduction of the cell number to zero after 2 (*C. albicans*) or 4 h (*C. glabrata*), while in terms of MIC concentration *C. glabrata* was more sensitive (complete elimination of living cells occurred after 4 h).

In this study we also aimed to check the effect of EOs on the cell wall and cell membrane. In the case of investigating the effect on cell wall using sorbitol assay, the most puzzling results were obtained for cinnamon oil, as we observed a decrease in MIC value in a medium supplemented with sorbitol as osmoprotectant, instead of the expected increase. The main compound of cinnamon oil is cinnamaldehyde, which is thought to inhibit the activity of β-1,3-glucan and chitin synthase [28], two enzymes responsible for producing fungal cell wall components. The fungal cell wall is a dynamic structure that protects the cell from changes in osmotic pressure and other environmental stresses, while allowing the fungal cell to interact with its environment. The structure and biosynthesis of a fungal cell wall is unique to the fungi, and is therefore an excellent target for the development of antifungal drugs [29]. Our results revealed that the effect of cinnamon oil, as well as cinnamaldehyde, in both sorbitol- and glycerol-supplemented mediums was the opposite to what we assumed: MIC values decreased up to 16-fold. Sorbitol/glycerol is an agent stabilizing the osmotic pressure of the cell, and thus the MIC values of strong cell wall inhibitors are believed to be increased in their presence. On the other hand, high osmolarity causes osmotic stress and results in metabolism changes. The most predominant physiological effect is the production of intracellural glycerol to counterbalance the external osmotic pressure. The production of glycerol is a highly glucose-consuming process. As a result, cells exhibit limited activity of β-1,3-glucan synthase as well as chitin synthase. Sorbitol is therefore a factor that causes slight cell stress, which may cause the inhibition of cell growth in the presence of some nonspecific cell wall inhibitors [30,31]. As both cinnamaldehyde and sorbitol influence β-1,3-glucan synthase as well as chitin synthase activity, the decrease in MIC value for cinnamon oil in the presence of this osmoprotectant is explainable. In the case of the remaining oils, the effect on the cell wall was not so evident. A two-fold increase of the MIC value in the presence of sorbitol in the medium was observed for basil oil (for both *C. albicans* and *C. glabrata*). The investigations of basil oil’s mode of anticandidal action are not very abundant, so it is hard to find a correlation concerning its effect on the cell wall. According to Cardoso’s group basil oil, as well as geraniol, contributes to marked cell wall thickening (results obtained on the basis of transmission electron microscopy images) [32]. On the other hand, Kaya et al. reported that *O. basilicum* extracts possess antibacterial activity by causing bacterial cell wall degradation [33]. The effect of *P. graveolens* EO is also not obvious. Essid and coworkers noted a two-fold increase of MIC of this EO in a medium supplemented with sorbitol, thereby suggesting its influence on the cell wall [7]. In our research, an increase in MIC value was not observed for *C. albicans* isolate but a two-fold increase was noted for *C. glabrata*; this does not give us an unambiguous answer about the influence of the oil on the cell wall. Overall, the changes in minimum inhibitory concentrations for the tested EOs were not significant in comparison to other well-known cell wall inhibitors; thus, we claim that the cell wall is a target of secondary importance.

Further research revealed that thyme, lemon, and clove oils influence the cell membranes. Up to a 16-fold increase in MIC value was observed for lemon oil for the *C. glabrata* strain, even higher than for amphotericin B (8-fold increase for both species), which binds the ergosterol found in lipid bilayer membranes. In the case of *C. albicans*, the change was not so noticeable and reached a 2-fold increase. Additionally, we suggest that the *C. glabrata* cell membrane may be more susceptible to the action of some EOs, as can be seen (apart from the mentioned lemon EO) in the case of geranium and clove oil (8- and at least 4-fold increase, respectively, while for *C. albicans* no increase was observed for geranium oil and for clove oil there was at least a 2-fold increase). The 4-fold increase of thyme oil MIC values in both tested strains also indicates its significant role in cell membrane disintegration. On the other hand, cinnamon oil does not seem to affect the cell membrane, as MIC values for this EO in the presence or absence of ergoesterol were unchanged for both species. Results obtained by other researchers also indicate the cell membrane as a potential molecular target of some EOs or their components. This can be, for instance, confirmed by Thakre and co-workers, who suggested that limonene inhibits *C. albicans* growth by cell wall/membrane damage [34]. A similar statement was made by Xu et al. pertaining to clove oil [35]. The influence of thyme oil on the cell membrane was observed by Rajkowska and co-workers, who noticed an up to 32-fold increase of MIC for this oil in ergosterol assay toward *C. albicans* ATCC 10231 [36]. We also evaluated cell membrane disintegration by the measurement of potassium ion leakage. In the case of clove, the leakage was dose-dependent. The highest efflux was noticed in the case of thyme oil, but it was not proportional to the concentration used (probably the concentration of 1 × MIC was large enough to cause the highest outflow of ions, so a further increase did not result in additional changes). Interestingly, a relatively high efflux of potassium ions was caused by cinnamon oil, compared to a very low one caused by lemon oil, which is not in agreement with the results of the ergosterol assay.

In our previous research we evaluated *CDR1* and *CDR2* genes expression level coding for drug efflux transporters in *C. glabrata* clinical isolates [37]. Taking into consideration isolates with significantly elevated gene expression levels according to the isolate susceptible to fluconazole, the MIC values for the tested oils were in the range: thyme < 0.005–0.31, lemon < 0.005–0.08, geranium 0.04–1.25, cinnamon < 0.002–0.125, basil < 0.005–0.63 and clove 0.04–1.25. If most constituents of EOs were substrates for these transporters, the assumed values of MIC concentrations would rather reach the highest range. In our experiment we observe that the MIC values of all tested EOs were not significantly different in the group of strains that upregulate drug pumps. Thus, we suggest that components of EOs do not necessarily have to be the substrates for CDR1p and CDR2p transporters.

We also considered the possibility of synergism between common antifungals and essential oils. Ahmad et al. estimated that thymol and its isomer carvacrol possess synergistic action with fluconazole toward some isolates of the genus *Candida* [38]; however, the calculated FIC index was in most cases 0.5, indicating small synergistic action. Guo showed a synergistic interaction between thymol and fluconazole or amphotericin B [39]. In our study the interaction between fluconazole and thyme oil was assigned as indifferent; however, thymol was not the predominant compound of the tested thyme oil. We found the possibility of interaction of amphotericin B with cinnamon or geranium oil. Amphotericin B is still a very powerful antifungal drug and resistance is rarely seen. However, at the same time it is a toxic compound used only in advanced fungemia, so any dosage reduction relating to the simultaneous usage of plant extract could be useful in antifungal chemotherapy.

Molecular switching between yeast, pseudohyphae, and hyphae phenotype is considered one of the most important virulence factors of *C. albicans* as it enables the evasion of the host immune system and rapid infection establishment. Therefore, factors inhibiting hypha formation are considered interesting leads that could help in the prevention of invasive fungemia. In our study we showed that most of the tested EOs have the potential for *C. albicans* hypha formation inhibition. In this matter our results are convergent with those obtained by Braga (inhibition of hypha formation by thymol) [40], Pozzatti (inhibition of germ tube formation by basil, cinnamon, and thyme oils) [41], and Zore (inhibition of germ tube formation by geranium oil) [42].

The findings reported in this paper indicate the high antifungal potential of some essential oils against *C. albicans* and *C. glabrata* clinical isolates. This result is very interesting from the point of view of their potential use as an alternative for conventional treatment. Additionally, experiments confirming the possibility of a synergistic effect with amphotericin B allow us to conclude that EOs can be used as a supplement for traditional chemotherapy. The range of effective concentrations would allow for their use in treatment, e.g., in topical applications. Modes of EOs action undoubtedly require further clarification but it is commonly known that a rich mixture of compounds may cause many simultaneous cellular effects. Therefore, future experiments will focus on investigations of the mode of action of single compounds. Additionally, it is of great importance to establish which particular components of a mixture exert a synergistic effect with common antifungal drugs.

## 4. Materials and Methods

### 4.1. Determination of Minimum Inhibitory Concentration (MIC) and Minimum Fungicidal Concentration (MFC)

The study included the evaluation of the activity of essential oils obtained from Pollena Aroma Company ((Nowy Dwór Mazowiecki, Poland). Antifungal susceptibility testing of *C. albicans* and *C. glabrata* clinical isolates was performed according to the NCCLS reference microdilution method. Serial two-fold dilutions of the tested substances were prepared in RPMI 1640 medium (Sigma Aldrich, St. Louis, MO, USA) buffered to pH 7.0 with MOPS buffer (3-*N*-morpholinopropanesulfonic acid, EMD Chemicals, Gibbstown, NJ, USA) in 96-well microtiter plates in a final volume of 100 µL. Cinnamon oil was diluted 10-fold in DMSO (Sigma Aldrich). The final concentrations of the oils were in the range 0.005–2.5% (*v*/*v*) and 0.002–0.125% (*v*/*v*) for cinnamon oil. The final concentration of the solvent did not exceed 2.5% for DMSO, and did not influence the growth of yeast. Suspensions of the microorganisms were prepared by taking one loop of pure culture into sterile water and adjusting the optical density to 0.1 at 660 nm wavelength before further 50-fold dilution in an RPMI 1640 medium resulting in 2 × 10^4^ CFU/mL. One hundred microliters of such suspension were inoculated to each well of the microtiter plate, leaving a drug-free column as sterility controls. Plates were incubated for 24 h at 37 °C. MIC values were read visually as the first concentration where no growth was observed.

Additionally, minimal fungicidal concentrations (MFC) were investigated. A small aliquot of suspension (around 5 µL) from each well was transferred using a 48-well stamp to YPD (Yeast extract Peptone Dextrose) (A&A Biotechnology, Gdynia, Poland) agar plates and incubated for 24 h at 37 °C. Concentrations where no growth was observed were assigned as MFC.

### 4.2. Time Kill Assay

From the overnight culture (16–18 h) on YPD agar plates, a cell suspension was prepared in sterile water and the optical density at OD_660_ was adjusted to about 0.1 (corresponding to 10^6^ cells per 1 mL of suspension). The inoculum was then diluted 10-fold in RPMI 1640 medium and treated with selected essential oils with the following concentrations: thyme—0.04%, lemon 0,16%, geranium—0.16%, basil 0.16%, clove—0.625% and for cinnamon oil cinnamon—0.016 or 0.031%, [*v*/*v*]. The suspensions of cells under the oils’ influence were first vigorously shaken and then incubated at 37 °C for 0.5, 2, 4, 8, and 24 h. After the appropriate time of incubation, 1 mL of each suspension was centrifuged (3 min, 5000 RPM) and resuspended in PBS (Sigma Aldrich, St. Louis, MI, USA) pH 7.4 (phosphate-buffered saline). Ten-fold serial dilutions were prepared and 100 µL of each were inoculated on YPD agar plates. Plates were incubated for 24 h at 37 °C. Colony-forming units in the range 30–300 were counted and the number of cells in 1 mL (CFU/mL) was calculated.

### 4.3. Sorbitol Assay Effect of Essential Oils on the Cell Wall of C. albicans and C. glabrata

The effect of essential oils on the cell wall of *C. albicans* and *C. glabrata* strains was analyzed using a medium with the addition of sorbitol as an osmoprotectant. The final concentration of sorbitol (Sigma Aldrich, St. Louis, MI, USA) in each well was 0.8 M. The assay was performed by the microdilution method in 96-well plates in a manner like in the “Determination of Minimum Inhibitory Concentration”. Plates were incubated for 48 h at 37 °C. Sorbitol acted as the fungal cell wall osmotic protective agent so the MIC values in a medium containing an agent acting against the cell wall in the presence of sorbitol are supposed to be higher than in a medium without the addition of sorbitol, confirming essential oils’ components’ interactions with cell wall building elements. Additionally, MIC values in the presence of cinnamaldehyde and sorbitol were checked to compare with the activity of cinnamon oil. Also, MFC values were evaluated according to the method described in “Determination of Minimum Inhibitory Concentration (MIC) and Minimum Fungicidal Concentration (MFC)”.

### 4.4. Ergosterol Binding Assay-MIC Value Determination in the Presence of Ergosterol

To assess whether the product binds to the fungal membrane sterols, MIC values with the addition of an exogenous source of ergosterol and without ergosterol were evaluated. Medium with ergosterol was prepared at the time of the test. To this end, ergosterol powder (Sigma Aldrich) was dissolved in DMSO (no more than 4% of final volume) and Tween 80 (Sigma Aldrich) (no more than 1% of final volume), heated to a temperature of 55 °C and mixed intensively. The solution was added to RPMI 1640 medium through a 0.2-µm filter (Merck Millipore, Burlington, MA, USA). Plates were prepared according to the method described in “Determination of Minimum Inhibitory Concentration”. The final concentration of ergosterol in each well was 100 µg/mL. Plates were incubated for 24–48 h at 37 °C. Amphotericin B (Sigma Aldrich) was used as a control as it is known to affect the membrane ergosterol. Investigations were carried out in duplicate. MIC values were read as the lowest concentrations where no growth of yeast was observed. If the mechanism of the selected essential oil action is associated with membrane sterols, the MIC in the presence of ergosterol is supposed to be higher than in a medium without ergosterol, thus this binding assay reflected the ability of the compound to bind with the ergosterol. Additionally, MFC values were evaluated according to the method described in “Determination of Minimum Inhibitory Concentration (MIC) and Minimum Fungicidal Concentration (MFC)”.

### 4.5. Potassium Efflux

*C. albicans* ATCC 10231 strain was grown overnight in Sabouraud medium (Oxoid, Basingstokee, UK) (150 RPM, 30 °C). Cell suspension was centrifuged, washed twice with Milli-Q water. Optical density (OD_660_) was adjusted to 1.0 in Milli-Q water. Cells were treated with EOs at concentrations corresponding to 1 × MIC, 2 × MIC and 4 × MIC (1 × MIC for the oils were as follows: thyme 0.625%, lemon 0.625%, geranium 1.25%, cinnamon 0.016%, basil 0.31%, clove 1.25%. The presented MIC values are higher than in the case of studying time kill assay, because experiments were performed about one year later, which resulted in a significant decrease in the oils’ activity). At the same time, solutions of EOs of the same concentrations were prepared as controls of potassium ion content in oils. The cells were vigorously shaken for 10 min at room temperature. The samples were centrifuged (3000 RPM, 5 min) and the supernatant was transferred to new tubes. The ion potassium concentration was measured with a flame ionizing detector BWB-1 (BWB Technologies Ltd., Newburye, UK).

### 4.6. Yeast to Mycelia Morphological Transformation

*C. albicans* ATCC 10231 strain was grown overnight in Sabouraud medium (150 RPM, 30 °C). Cells were washed twice with sterile water and the cell number concentration was adjusted to 10^6^ cells per mL in Lee medium [43], which induces the growth of hypha forms. Cell suspensions were treated with EOs tested at concentrations equal to MIC or ½ × MIC and were incubated for 2 h or 24 h, respectively. After an appropriate time, the number of mycelium forms was counted in a Thoma cell counting chamber and compared to a control not treated with any EOs.

### 4.7. Synergy between Antifungals and Essential Oils

Possible synergistic action of common antifungal agents in combination with the essential oils was determined by the checkerboard method. After overnight culture (16–18 h) on YPD agar plates, colonies of the reference strains (*C. albicans* ATCC 10231, *C. glabrata* DSM 11226) were suspended in sterile water. Optical density (OD_660_) was adjusted to about 0.1 (corresponding to 10^6^ cells in 1 mL) and the inoculum was diluted 50-fold to obtain a cell density of 2 × 10^4^ cells per 1 mL of suspension. A gradient of antifungal chemotherapeutic was established along the horizontal axis and essential oil along the vertical axis. The first row contained only the gradient of chemotherapeutic and the tenth column gradient of the natural product. One hundred microliters of the prepared suspension were inoculated to each well of the plate. Plates were incubated for 24 h at 37 °C. The MIC values of the compound alone or in combination were read visually. ΣFIC (fractional inhibitory concentrations) was determined according to the equation ΣFIC = FIC A + FIC B, where FIC A is the MIC of a medicament in combination/MIC of the drug alone, FIC B is the MIC of the second compound in combination/MIC of the compound alone. The combination is considered synergistic when the ΣFIC is ≤ 0.5, indifferent when the ΣFIC is > 0.5 to < 4, and antagonistic when the ΣFIC is ≥ 4.

### 4.8. GC × GC MS Analysis of Selected Essential Oils

Analysis of the chemical composition of the selected essential oils was performed by using a comprehensive two-dimensional chromatography with Agilent 7890A equipment (Agilent Technologies, Palo Alto, CA, USA) and the time-of-flight mass spectrometry Pegasus IV detector (LECO Corp., St. Joseph, MI, USA). Analysis was provided using a conventional Equity 1 column (30 m × 0.25 mm i.d, 0.25 µm film thickness, SUPELCO Co., Bellefonte, PA, USA), while the secondary fast column was a SGWAX (2 m × 0.10 mm i.d., 0.10 µm film thickness, SUPELCO Co., Bellefonte, PA, USA). The operational conditions for the first oven were: 40 °C (3.5 min), temperature rise 7 °C/min; 250 °C (9.3 min), second oven 70 °C (3.5 min), temperature rise 7 °C/min; 250 °C (9.3 min); detector temperature 250 °C. The injector was working in splitless mode at temperature 250 °C. Helium (N6.0 grade; Linde AG, Munich, Germany), was used as a carrier gas with a flow rate of 1 mL/min, ionization energy of 70 eV, and ion source temperature of 250 °C in the *m*/*z* range 40–400. One milliliter of each essential oil was transferred to a 20-mL vial and sealed with a cap with PTFE-lined silicone septum. Headspace solid-phase microextraction (HS-SPME) was used to extract volatile compounds. A divinylbenzene/carboxen/polydimethylsiloxane (DVB/CAR/PDMS) SPME fiber of 50/30 μm thickness and 2 cm length (Sigma-Aldrich) was used. The samples were kept at 40 °C for 5 min and agitated with a magnetic stirrer (450 rpm). Extraction was conducted at 40 °C for 25 min. After the extraction, the fiber was transferred to the injector of a gas chromatograph for thermal desorption of the analytes for 2 min. Identification of components was carried out using a system equipped with commercial libraries (NIST 2011 (NIST, Gaithensburg, MD, USA) and Wiley 11 (WILEY-VCH, Hoboken, NJ, USA) and by comparison of linear temperature-programmed retention indices (RI). The mixture of C_7_–C_30_ n-alkanes (Sigma-Aldrich, Saint Louis, MO, USA) was used for the calculation of RI. A semi-quantitative analysis (expressed as percentages of each component) was carried out by peak area normalization measurements without correction factors. Each sample of essential oil was analyzed in triplicate. The total number of analyzes was 18.

## 5. Conclusions

The results from this study revealed that some EOs could be used as an alternative agent for treatment of infections caused by *Candida* spp. *Pelargonium graveolens*, but also *Cinnamomum cassia* essential oils exhibited synergistic activity with amphotericin B, which allows us to conclude that these EOs can be used as a supplement for traditional chemotherapy. Most of the oils were effective in inhibition of mycelia morphological transformation. Our research revealed also that cell membrane seems to be the most important target of ingredients of investigated oils.

## Figures and Tables

**Table 1:** Number of isolates with exact MIC value of the selected essential oil in the group of 183 *C. albicans* clinical isolates.

	Thyme		Lemon		Geranium		Basil		Clove		Cinnamon		
% (*v*/*v*)	mg/mL	*n*	mg/mL	*n*	mg/mL	*n*	mg/mL	*n*	mg/mL	*n*	% (*v*/*v*)	mg/mL	*n*
<0.005	<0.046	30	<0.043	21	<0.044	1	<0.048	24	<0.053	2	<0.002	<0.021	114
0.005	0.046	3	0.043	3	0.044	0	0.048	0	0.053	0	0.002	0.021	9
0.010	0.092	28	0.085	13	0.089	6	0.096	1	0.105	6	0.004	0.041	12
0.020	0.183	14	0.171	12	0.177	8	0.191	6	0.210	8	0.008	0.082	9
0.040	0.367	20	0.341	21	0.355	17	0.383	7	0.420	17	0.016	0.165	12
0.080	0.734	31	0.682	20	0.710	30	0.766	21	0.840	29	0.031	0.321	16
0.160	1.467	22	1.365	20	1.419	47	1.531	30	1.680	35	0.062	0.644	10
0.313	2.866	26	2.666	31	2.772	40	2.991	55	3.281	29	0.125	1.288	1
0.625	5.731	9	5.331	23	5.544	28	5.981	26	6.563	29	0.250	2.575	0
1.250	11.463	0	10.663	12	11.088	6	11.963	13	13.125	20	0.500	5.150	0
2.500	22.925	0	21.325	5	22.175	0	23.925	0	26.250	8	1.000	10.300	0
>2.500	>22.925	0	>21.325	2	>22.175	0	>23.925	0	>26.25	0	>1.000	>10.3	0

**Table 2:** Number of isolates with exact MFC value of the selected essential oil in the group of 183 *C. albicans* clinical isolates.

	Thyme		Lemon		Geranium		Basil		Clove		Cinnamon		
% (*v*/*v*)	mg/mL	*n*	mg/mL	*n*	mg/mL	*n*	mg/mL	*n*	mg/mL	*n*	% (*v*/*v*)	mg/mL	*n*
<0.005	<0.046	11	< 0.043	12	<0.044	0	<0.048	19	<0.053	0	<0.002	<0.021	89
0.005	0.046	0	0.043	1	0.044	0	0.048	0	0.053	0	0.002	0.021	5
0.010	0.092	15	0.085	13	0.089	0	0.096	5	0.105	1	0.004	0.041	15
0.020	0.183	18	0.171	8	0.177	1	0.191	4	0.210	1	0.008	0.082	19
0.040	0.367	18	0.341	23	0.355	7	0.383	6	0.420	7	0.016	0.165	15
0.080	0.734	14	0.682	16	0.710	10	0.766	10	0.840	17	0.031	0.321	16
0.160	1.467	35	1.365	12	1.419	42	1.531	22	1.680	22	0.062	0.644	17
0.313	2.866	34	2.666	24	2.772	54	2.991	47	3.281	34	0.125	1.288	7
0.625	5.731	30	5.331	20	5.544	43	5.981	45	6.563	37	0.250	2.575	0
1.250	11.463	6	10.663	27	11.088	22	11.963	20	13.125	36	0.500	5.150	0
2.500	22.925	2	21.325	21	22.175	4	23.925	3	26.250	27	1.000	10.300	0
>2.500	>22.925	0	>21.325	6	>22.175	0	>23.925	2	>26.25	1	>1.000	>10.3	0

**Table 3:** Number of isolates with exact MIC value of the selected essential oil in the group of 76 *C. glabrata* clinical isolates.

	Thyme		Lemon		Geranium		Basil		Clove		Cinnamon		
% (*v*/*v*)	mg/mL	*n*	mg/mL	*n*	mg/mL	*n*	mg/mL	*n*	mg/mL	*n*	% (*v*/*v*)	mg/mL	*n*
<0.005	<0.046	15	<0.043	27	<0.044	0	<0.048	7	<0.053	2	<0.002	<0.021	43
0.005	0.046	0	0.043	0	0.044	0	0.048	0	0.053	0	0.002	0.021	1
0.010	0.092	6	0.085	5	0.089	0	0.096	1	0.105	0	0.004	0.041	5
0.020	0.183	7	0.171	5	0.177	2	0.191	5	0.210	1	0.008	0.082	5
0.040	0.367	7	0.341	8	0.355	7	0.383	0	0.420	10	0.016	0.165	5
0.080	0.734	13	0.682	3	0.710	17	0.766	11	0.840	12	0.031	0.321	7
0.160	1.467	16	1.365	18	1.419	20	1.531	14	1.680	16	0.062	0.644	5
0.313	2.866	9	2.666	8	2.772	19	2.991	20	3.281	11	0.125	1.288	5
0.625	5.731	3	5.331	2	5.544	9	5.981	15	6.563	10	0.250	2.575	0
1.250	11.463	0	10.663	0	11.088	2	11.963	3	13.125	13	0.500	5.150	0
2.500	22.925	0	21.325	0	22.175	0	23.925	0	26.250	1	1.000	10.300	0
>2.500	>22.925	0	>21.325	0	>22.175	0	>23.925	0	>26.25	0	>1.000	>10.3	0

**Table 4:** Number of isolates with exact MFC value (% *v*/*v*) of the selected essential oil in the group of 76 *C. glabrata* clinical isolates.

	Thyme		Lemon		Geranium		Basil		Clove		Cinnamon		
% (*v*/*v*)	mg/mL	*n*	mg/mL	*n*	mg/mL	*n*	mg/mL	*n*	mg/mL	*n*	% (*v*/*v*)	mg/mL	*n*
<0.005	<0.046	9	<0.043	21	<0.044	0	<0.048	4	<0.053	0	<0.002	<0.021	41
0.005	0.046	0	0.043	0	0.044	0	0.048	0	0.053	0	0.002	0.021	1
0.010	0.092	7	0.085	3	0.089	0	0.096	1	0.105	2	0.004	0.041	5
0.020	0.183	2	0.171	10	0.177	1	0.191	2	0.210	0	0.008	0.082	3
0.040	0.367	5	0.341	2	0.355	6	0.383	3	0.420	7	0.016	0.165	8
0.080	0.734	9	0.682	2	0.710	14	0.766	7	0.840	8	0.031	0.321	6
0.160	1.467	23	1.365	5	1.419	17	1.531	14	1.680	14	0.062	0.644	8
0.313	2.866	14	2.666	17	2.772	22	2.991	20	3.281	16	0.125	1.288	4
0.625	5.731	6	5.331	11	5.544	11	5.981	20	6.563	14	0.250	2.575	0
1.250	11.463	1	10.663	5	11.088	3	11.963	4	13.125	6	0.500	5.150	0
2.500	22.925	0	21.325	0	22.175	1	23.925	0	26.250	9	1.000	10.300	0
>2.500	>22.925	0	>21.325	0	>22.175	1	>23.925	1	>26.25	0	>1.000	>10.3	0

**Table 5:** MIC values of the tested oils in the presence and absence of sorbitol/ergosterol.

EO	*C. albicans* ATCC 10231			*C. glabrata* DSM 11226		
	RPMI	RPMI + Sorbitol	RPMI + Ergosterol	RPMI	RPMI + Sorbitol	RPMI + Ergosterol
thyme	0.62	0.08	2.50	0.31	0.16	1.25
lemon	0.62	0.62	1.25	0.08	0.08	1.25
geranium	1.25	1.25	0.62	0.16	0.31	1.25
cinnamon	0.016	<0.005	0.016	0.031	0.002	0.031
basil	0.31	0.62	0.31	0.31	0.31/0.62	0.16
clove	1.25	0.31	≥2.5	0.6	2.50	≥2.5
AmB	0.06		0.5	0.06		8

**Table 6:** MIC values for cinnamon oil and cinnamaldehyde in the presence and absence of sorbitol/glycerol.

Investigated Strain	RPMI 1640		RPMI + Sorbitol		RPMI + Glycerol	
	Cinnamon Oil	Cinnamaldehyde	Cinnamon Oil	Cinnamaldehyde	Cinnamon Oil	Cinnamaldehyde
*C. albicans* ATCC 10231	0.008	0.008	<0.002	<0.002		
*C. glabrata* DSM 11226	0.031	0.031	<0.002	<0.002		
*C. albicans* 412 CZD	0.016	0.016	<0.002	<0.002	0.008	<0.002
*C. glabrata* 342 CZD	0.031	0.031	<0.002	<0.002	<0.002	<0.002

**Table 7:** The content of hypha forms after EO treatment.

Sample	% of Mycelium Forms	
	After 2 h	After 24 h
Control	95	22
Thyme oil	0	0
Lemon oil	0	0
Geranium oil	0	0
Cinnamon oil	0	27
Basil oil	5	11
Clove oil	0	0

**Table 8:** Chemical composition of EOs tested obtained with GC × GC MS and content of each compound estimated from the area under the peak.

Compound	RI	RI_Lit_	Area under the Peak (%)					
			Thyme	Lemon	Geranium	Cinnamon	Basil	Clove
2-Amylfuran	1039	1040			0.01			
2-Bornanone	1125	1121					4.62	
2-methoxy-benzaldehyde	1167	1171				0.23		
Acetophenone	1026	1029				1.02		
Alloaromadendrene	1381	1386			0.02			
Anethole	1196	1190					0.82	
Anisaldehyde	1167	1171					0.46	
α-Pinene	948	948			3.44			
Aromadendrene	1380	1386				0.16		
Benzaldehyde	981	982				24.6		
Benzenepropanal	1189	1181				0.26		
Benzofuran	1019	1018				0.15		
Bergamotene	1432	1430				0.06	6.65	
Borneol	1135	1138				0.61		
Bornyl acetate	1273	1277				0.07		
Bornyl formate	1283	1275				0.32		
Bornylene	928	932			8.21			
Bourbonene	1331	1339			1.14	0.17	0.09	
β-Phenylethyl formate	1157	1157			0.54			
Cadinene	1442	1440	0.01				0.25	0.33
Calamenene	1543	1537			0.19			
Camphene	942	943	1.27	0.64	0.34	1.86	2.25	
Camphor	1125	1121	0.4				8.75	
Carene	1059	1055		0.02			0.06	
Carvacrol methyl ether	1241	1231	0.42	0.16				
Carveol	1208	1206						
Carvomenthene	989	987	0.08	0.4				
Caryophyllene	1499	1494	0.2					14.47
Cinnamaldehyde	1186	1189				42.79	0.87	0.19
Cinnamyl acetate	1370	1367				0.13		
cis-Geranyl acetate	1340	1352			0.31			
Citral	1169	1174		53.85	0.02		0.29	
Citronellol	1192	1179			11.94			
Citronellyl formate	1294	1300			13.2			
Citronellyl propionate	1400	1402			0.05			
Copaene	1227	1430			0.4	1.29	0.07	0.61
Cycloisosativene	1121	1125				0.26		
Cymene	1035	1042	1.14		2.52	2.48	2.68	0.1
Cymenene	1068	1073		0.51				
Decane	1009	1015						0.13
endo-Borneol	1131	1138					0.99	
epoxyocimene							0.39	
Estragole	1166	1172	0.02	0.11		0.16	6.18	0.06
Eucalyptol	1065	1059					35.44	
Eugenol	1399	1392						68.24
exo-Fenchol	1064	1062					3.04	
Farnesene	1455	1458					0.18	
Furfural	833	831				0.23		
Geraniol	1231	1228			2.99			
Geraniol formate	1353	1349			5.02			
Heptane	715	717						0.67
Herboxide second isomer	1036	1040			0.16			
Humulene	1591	1579	0.01		0.17			9.09
Isobornyl acetate	1281	1277					3.01	
Limonene	1020	1018	0.12	5.29				0.45
Limonene oxide	1027	1031	0.97					
Linalool	1082	1082	0.96		11.28			
Linalool oxide	1165	1164			0.1		5.64	
Menthol	1167	1164			0.01			
Methyl thymylether	1222	1231	0.67					
Methyleugenol	1366	1361					0.13	
Muurolene	1415	1419				0.31		
Myrcene	956	958	34.35	0.7	0.11		0.24	
Ocimene	994	993			0.49		3.2	
Octadecane	1812	1810		0.02				
Octen-3-ol	971	969	0.88					
Phellandrene	974	969	0.67	0.46	0.3			
Phenylethyl alcohol	1134	1136			2.66	0.69		
Phenylethyl formate	1261	1257				0.76		
Phytane	1749	1753						0.01
Pinene	947	948	2.61	0.05	0.04	1.02	1.36	
Piperitone	1152	1158		0.14				
p-Menth-2.8-dien-1-ol	1136	1140		0.33				
p-Menthone	1151	1148			17.85			
Rose oxide A	1120	1114			5.59			
Rose oxide B	1121	1114			1.93			
Sabinene	896	897	0.02				0.04	
Safrole	1329	1327				0.11		
Salicylaldehyde	1211	1203				2.57		
Styrene	887	883				8.45		
Sulcatone	939	938			0.15	0.47		
Terpinen-4-ol	1140	1137					0.15	
Terpinene	998	998	46.55	4.49	0.03		0.32	
Terpineol	1142	1137			0.91			
Terpinolene	1055	1052		4.57				
Tetrahydro geraniol	1138	1130			0.02			
Thymol	1266	1262	1.75	0.11				
Toluene	795	794						3.41
Others			7.88	27.18	7.85	8.77	11.82	2.25

RI—averaged RI for analyses. RI_Lit_—RI value for 100% PDMS stationary phase; data taken from NIST 2011 spectral library
